# Iron Triad-Based Bimetallic M–N–C Nanomaterials as Highly Active Bifunctional Oxygen Electrocatalysts

**DOI:** 10.1021/acsaem.4c00366

**Published:** 2024-05-02

**Authors:** Mahboob Alam, Kefeng Ping, Mati Danilson, Valdek Mikli, Maike Käärik, Jaan Leis, Jaan Aruväli, Päärn Paiste, Mihkel Rähn, Väino Sammelselg, Kaido Tammeveski, Steffen Haller, Ulrike I. Kramm, Pavel Starkov, Nadezda Kongi

**Affiliations:** † Department of Chemistry and Biotechnology, Tallinn University of Technology, Tallinn 12618, Estonia; ‡ Department of Materials and Environmental Technology, Tallinn University of Technology, Tallinn 19086, Estonia; § Institute of Chemistry, University of Tartu, Tartu 50411, Estonia; ∥ Institute of Ecology and Earth Sciences, University of Tartu, Tartu 50411, Estonia; ⊥ Institute of Physics, University of Tartu, Tartu 50411, Estonia; # Department of Chemistry, Catalysts and Electrocatalysts Group, Technical University of Darmstadt, Darmstadt 64287, Germany

**Keywords:** electrocatalysis, oxygen reduction, oxygen evolution, Zn–air batteries, M−N–C catalysts, nonprecious metal catalysts

## Abstract

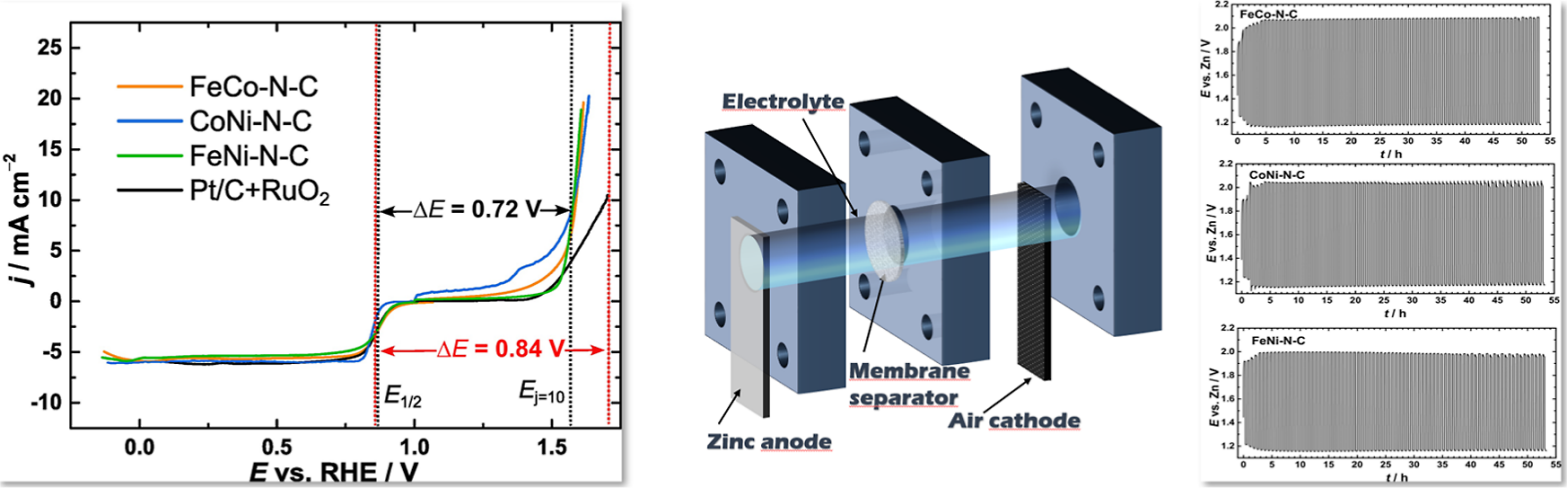

The use of precious metal electrocatalysts in clean electrochemical energy conversion and storage applications is widespread, but the sustainability of these materials, in terms of their availability and cost, is constrained. In this research, iron triad-based bimetallic nitrogen-doped carbon (M–N–C) materials were investigated as potential bifunctional electrocatalysts for the oxygen reduction reaction (ORR) and oxygen evolution reaction (OER). The synthesis of bimetallic FeCo–N–C, CoNi–N–C, and FeNi–N–C catalysts involved a precisely optimized carbonization process of their respective metal–organic precursors. Comprehensive structural analysis was undertaken to elucidate the morphology of the prepared M–N–C materials, while their electrocatalytic performance was assessed through cyclic voltammetry and rotating disk electrode measurements in a 0.1 M KOH solution. All bimetallic catalyst materials demonstrated impressive bifunctional electrocatalytic performance in both the ORR and the OER. However, the FeNi–N–C catalyst proved notably more stable, particularly in the OER conditions. Employed as a bifunctional catalyst for ORR/OER within a customized zinc–air battery, FeNi–N–C exhibited a remarkable discharge–charge voltage gap of only 0.86 V, alongside a peak power density of 60 mW cm^–2^. The outstanding stability of FeNi–N–C, operational for about 55 h at 2 mA cm^–2^, highlights its robustness for prolonged application.

## 1 Introduction

To meet the growing demand for renewable energy, it is necessary to significantly increase the installed power of renewable energy sources. However, the intermittent nature of energy production from these sources will require the development of alternative energy-storage solutions to ensure a reliable and stable energy supply.^1^ Metal–air batteries offer high energy density but require advanced catalyst materials to ensure efficient and reversible electrochemical reactions for optimal performance and durability.^2−4^ In this domain, transition metal and nitrogen-codoped carbon (M–N–C) catalysts offer a more significant promise from a sustainable electrocatalysis perspective. M–N–C materials are composed of earth-abundant and cost-effective elements; hence, they may reach electrocatalytic performances that can outperform those of precious metal-based catalysts under distinct conditions.^5^ In this context, the application of iron triad metals (Fe, Co, and Ni) seems to be very promising based on their low cost, earth abundance, and high performance in fuel cells and metal–air batteries.^6−8^

There has been a lot of research on using mono- or bimetallic M–N–C catalysts for various reactions like the hydrogen evolution reaction (HER),^9−11^ oxygen reduction reaction (ORR),^12−16^ oxygen evolution reaction (OER),^17−19^ and CO_2_ reduction reactions.^10,20−23^ Variation of synthetic conditions results in the formation of nitrogen-coordinated metal species (M–N_*x*_ sites) and metal alloy, metal nitride, metal carbide, or metal oxide nanoparticles that are embedded in nitrogen-doped carbon layers.^24−26^ In recent years, research has demonstrated that certain species and their combinations in M–N–C materials have exhibited promising electrocatalytic activity in alkaline electrolytes.^27,28^ The use of bimetallic catalysts has been explored as an effective approach to enhance the bifunctional performance of M–N–C electrocatalysts.^29^ This approach aims to diversify the active sites available for electrocatalytic reactions, ultimately leading to improved performance compared to using a single metal.^9,30−33^ Bimetallic M–N–C materials have been found to have unique electronic and geometric properties, which can lead to improved catalytic activity.^34−40^ Moreover, the presence of metal oxides in the composition of multimetallic M–N–C can lead to improved stability and higher active-site densities specifically for the OER.^34,41−43^

The activity and stability of bimetallic M–N–C catalysts depend on the combination of metals used, the pH regime, temperature, and potential window during application. Some studies have shown that different faces of carbon-entrapped FeCo nanoalloys are active in ORR electrocatalysis and FeNi nanoalloy particles have been demonstrated as a bifunctional ORR/OER electrocatalyst.^29,44−46^ The choice of precursors and the method of preparation can also influence the electrocatalytic properties of the M–N–C materials.^18,47,48^ It is important to understand which sites of the catalysts contribute to the activity for a specific reaction and under what conditions these catalysts are most stable. Despite the potential benefits of bimetallic catalysts, the fabrication methods currently available have not been systematically explored, and hence, there is still a lot of research that needs to be done in this area to improve the scalability, reproducibility, and efficiency.

In this study, we aim to address the challenge of identifying optimal combinations of transition metals for the fabrication of M–N–C materials that incorporate carbon-embedded bimetallic nanoparticles of the iron triad. To accomplish this, we introduce a novel synthesis strategy involving the use of an electron-rich benzimidazole-derived organic ligand 1*H*-benzo[*d*]imidazole-5,6-diol.^49−52^ This ligand is designed to provide nitrogen- and oxygen-containing sites that facilitate the effective incorporation of various metals at the initial stage of metal–organic material (MOM) formation. The resulting iron triad MOM-derived materials are characterized and evaluated for their electrocatalytic activity in the ORR and the OER in alkaline media. The purpose of this work is to provide a new and more efficient approach for the design and development of bimetallic M–N–C-type catalysts with superior electrocatalytic activity and stability.

## 2 Experimental Section

### 2.1 Synthesis of Bimetallic MOMs

#### 2.1.1 FeCo MOM

The 1*H*-benzo[*d*]imidazole-5,6-diol ligand was prepared as previously reported.^50^ A solution of FeCl_3_·6H_2_O (1.35 g, 5.00 mmol, 1.0 equiv) and CoCl_2_·6H_2_O (1.19 g, 5.00 mmol, 1.0 equiv) in water (15 mL) was added dropwise into a mixture of 1*H*-benzo[*d*]imidazole-5,6-diol (3.00 g, 19.98 mmol, 4.0 equiv) in 25% aq NH_3_/DMF/EtOH (4 mL, 10 mL, 10 mL), and the resulting solution was left to stir at RT. After 24 h, it was filtered, washed with EtOH, and dried to give the desired material as a black solid.

#### 2.1.2 CoNi MOM

A solution of CoCl_2_·6H_2_O (1.61 g, 6.77 mmol, 1.0 equiv) and NiCl_2_·6H_2_O (1.61 g, 6.77 mmol, 1.0 equiv) in water (15 mL) was added dropwise into a mixture of 1*H*-benzo[*d*]imidazole-5,6-diol (4.07 g, 27.12 mmol, 4.0 equiv) in 25% aq NH_3_/DMF/EtOH (4 mL, 10 mL, 10 mL), and the resulting solution was left to stir at RT. After 24 h, it was filtered, washed with EtOH, and dried to give the desired material as a black solid.

#### 2.1.3 FeNi MOM

A solution of FeCl_3_·6H_2_O (2.25 g, 8.32 mmol, 1.0 equiv) and NiCl_2_·6H_2_O (1.97 g, 8.30 mmol, 1.0 equiv) in water (20 mL) was added dropwise into a mixture of 1*H*-benzo[*d*]imidazole-5,6-diol (5.00 g, 33.30 mmol, 4.0 equiv) in 25% aq NH_3_/DMF/EtOH (5, 15, 15 mL), and the resulting solution was left to stir at RT. After 24 h, it was filtered, washed with EtOH, and dried to give the desired material as a black solid.

### 2.2 Preparation of MOM-Derived M–N–C Catalysts

Synthesized MOMs were placed into a ceramic boat and heat treated by flash carbonization in a quartz tube furnace at 900 °C for 2 h in a nitrogen atmosphere (rapid heating, rapid cooling, and temperature ramping at 50 °C min^–1^). Carbonized materials were suspended in 0.5 M HNO_3_, stirred for 8 h at 50 °C, washed in Milli-Q water, filtered, and recarbonized under N_2_ at the same temperature (900 °C for 2 h) to give the final catalyst materials. Synthesized M–N–C electrocatalysts were designated as FeCo–N–C, CoNi–N–C, and FeNi–N–C. The data presented in Table S1 provides a summary of the yields obtained at each stage of the synthesis, enabling the reader to assess the efficiency and effectiveness of the postsynthetic treatment methods utilized in this study.

### 2.3 Physical Characterization

Transmission electron microscopy (TEM) measurements were performed using a JEOL-2200FS field emission gun (FEG) (S) TEM equipped with a Schottky FEG, operating at an accelerating voltage of 200 kV. The catalyst materials were dispersed in 2-propanol and sonicated for 10 min to prepare the TEM samples. The resulting suspension was pipetted on a 200-mesh copper grid covered by a carbon film. Scanning electron microscopy (SEM) measurements were performed using a Zeiss Ultra-55. In-lens secondary electron detection at a 4 kV accelerating voltage was used. X-ray photoelectron spectroscopy (XPS) measurements were performed by a SCENTA SES-100 spectrometer equipped with a 300 W nonmonochromatic Mg Kα X-ray source (incident energy = 1253.6 eV) and an electron takeoff angle of 90°. During XPS spectra collection, the analysis chamber pressure was below 1.3 × 10^–8^ mbar. Step sizes of 0.5 and 0.1 eV were used for collecting survey and high-resolution XPS spectra, respectively. For the XPS measurements, the catalyst powders were fixed on carbon tape. The X-ray diffraction (XRD) patterns for the samples were recorded on a Bruker D8 ADVANCE diffractometer using Cu Kα radiation and a silicon strip line detector. Scanning steps were 0.013° 2θ from 5 to 90°, 2θ, and the total counting time was 173 s/step. Low-temperature nitrogen adsorption–desorption analysis was done at the boiling temperature of nitrogen (77 K) using a NOVAtouch LX2 instrument (Quantachrome Instruments). Prior to nitrogen physisorption measurements, the samples were dried for 12 h in a vacuum at 150 °C. The specific surface area (*S*_BET_) of catalyst materials was calculated from N_2_ adsorption–desorption isotherms corresponding to the BET theory in the *P*/*P*_0_ interval of 0.02–0.2, and the total pore volume (*V*_tot_) was calculated at a *P*/*P*_0_ of 0.97. The pore size distribution and specific surface area were calculated from N_2_ physisorption isotherms using a quenched solid density functional theory equilibrium model for slit-type pores. All calculations were done using TouchWin 1.11 software (Quantachrome Instruments). For microwave plasma atomic emission spectroscopy (MP-AES), the samples were dissolved with Anton Paar Multiwave PRO microwave digestion system using NXF100 digestion vessels (PTFE-TFM liner) in an 8 N rotor before analysis. Ten milligrams of the sample were weighed into PTFE vessels into which 4 mL of 69% HNO_3_ and 2 mL of H_2_O_2_ were sequentially and slowly added. After the initial reaction had subsided, the vessels were capped and digested in the microwave unit at 240 °C and 45–50 bar pressure. After digestion, the samples were diluted using 2% HNO_3_ (prepared from 69% HNO_3_) to a final concentration of around 4 mg L^–1^ and analyzed using an Agilent MP-AES 4210. Iron, cobalt, and nickel were measured at 371.993, 340.512, and 352.454 nm, respectively.

### 2.4 Electrochemical Measurements

The electrocatalytic activity of synthesized catalyst materials was assessed by using a multichannel potentiostat/galvanostat (PGSTAT M204, Metrohm-Autolab, Utrecht, The Netherlands) controlled by NOVA 2.1.6 software. A five-neck electrochemical glass cell was used in all of the rotating disk electrode (RDE) experiments. The glassy carbon (GC) electrode with a diameter of 5 mm mounted into a Teflon holder served as a working electrode. Carbon rods and a reversible hydrogen electrode (RHE) were used as counter and reference electrodes, respectively. 5 mg of the catalyst powder was ultrasonically dispersed in 100 μL of 0.05 wt % Nafion solution in 2-propanol and deposited onto the GC surface to yield a catalyst loading of 0.50 mg cm^–2^. For comparison, commercial 20% Pt/C (E-TEK, loading of 0.10 mg cm^–2^) was used for the ORR, while RuO_2_ was used as a reference material for the OER (Alfa Aesar, loading of 0.12 mg cm^–2^).

#### 2.4.1 Oxygen Reduction Reaction

First, cyclic voltammetry (CV) experiments were performed to obtain a stable catalyst surface. Catalysts were cycled at least five times in argon-saturated 0.1 M KOH electrolyte solution between −0.2 and 1.1 V vs RHE at 50 mV s^–1^. Background CV curves were recorded at 10 mV s^–1^. Pt/C benchmark was cycled between 0.1 and 1.4 V vs RHE, and the CO-stripping voltammetry was performed to obtain a clean Pt surface.^53^ The ORR experiments were performed in an O_2_-saturated electrolyte, and RDE polarization curves were recorded at a potential scan rate (ν) of 10 mV s^–1^ at different electrode rotation speeds (360, 610, 960, 1600, 1900, and 3100 rpm). The background current was then subtracted from the RDE data to eliminate capacitive current contribution. The data obtained from RDE polarization curves were analyzed by the Koutecky–Levich (K–L) equation. To evaluate the efficiency of the catalyst materials in the ORR, the onset potential (*E*_on_, potential at *j* = −0.1 mA cm^–2^) and half-wave potential (*E*_1/2_, potential at *j* = −3 mA cm^–2^) values were calculated at 1600 rpm.

To explore the ORR stability of bimetallic M–N–C catalyst materials, the electrode potential was cycled 5000 times between 0.2 and 1.2 V vs RHE in an O_2_-saturated electrolyte at 200 mV s^–1^ (rotated at 1600 rpm) and RDE polarization curves at 1600 rpm before and after stability tests were compared.

#### 2.4.2 Oxygen Evolution Reaction

To obtain OER data, LSV curves were recorded at 1600 rpm in the 0.1 M KOH electrolyte using a scan rate of 10 mV s^–1^ in a potential window of 1.0–1.8 V vs RHE. Before the measurement, the electrode was cycled 40 times at the scan rate of 200 mV s^–1^ to activate the material. Electrochemical impedance spectroscopy (EIS) was performed to obtain *i*R-compensated potentials. Stability tests were conducted through the long-term potential cycling (5000 cycles) of the modified electrodes. For ORR, the cycling was performed at 1600 rpm within the potential range from +0.2 to +1.2 V vs RHE. In the case of the OER, the cycling range was from +1 to +1.8 V vs RHE.

#### 2.4.3 Zinc–Air Battery Tests

For ZAB tests, the catalyst inks were prepared by suspending 8 mg of the catalyst powder in 25 μL of 0.5% Nafion solution, 83 μL of 2-propanol, and 142 μL of Milli-Q water. This mixture was subjected to sonication for 1 h to achieve homogeneity. For the preparation of the air cathode, a circular section of the gas diffusion layer (GDL), specifically Freudenberg GDL H23C9, was modified with 39.5 μL of the ink, covering an area ranging from 0.75 to 0.79 cm^2^. In the anode assembly, a 0.25 mm-thick zinc plate underwent polishing with a 1 μm diamond particle slurry to eliminate oxides. The assembly involved the separation of the electrodes by a Celgard 5550 membrane.

The measurement of polarization curves was conducted by discharging the potential under a sweep rate of 1 mV s^–1^ in an alkaline electrolyte (6 M KOH + 0.2 M zinc acetate). To assess stability, a cycling protocol of 40 min charging and discharging cycles was iterated 80 times. After the cycling, a second polarization curve was acquired under the aforementioned conditions, thereby enabling the evaluation of the extent of activity depletion within the battery.

## 3 Results and Discussion

### 3.1 Synthesis of Materials and Physical Characterization

**Scheme 1:**
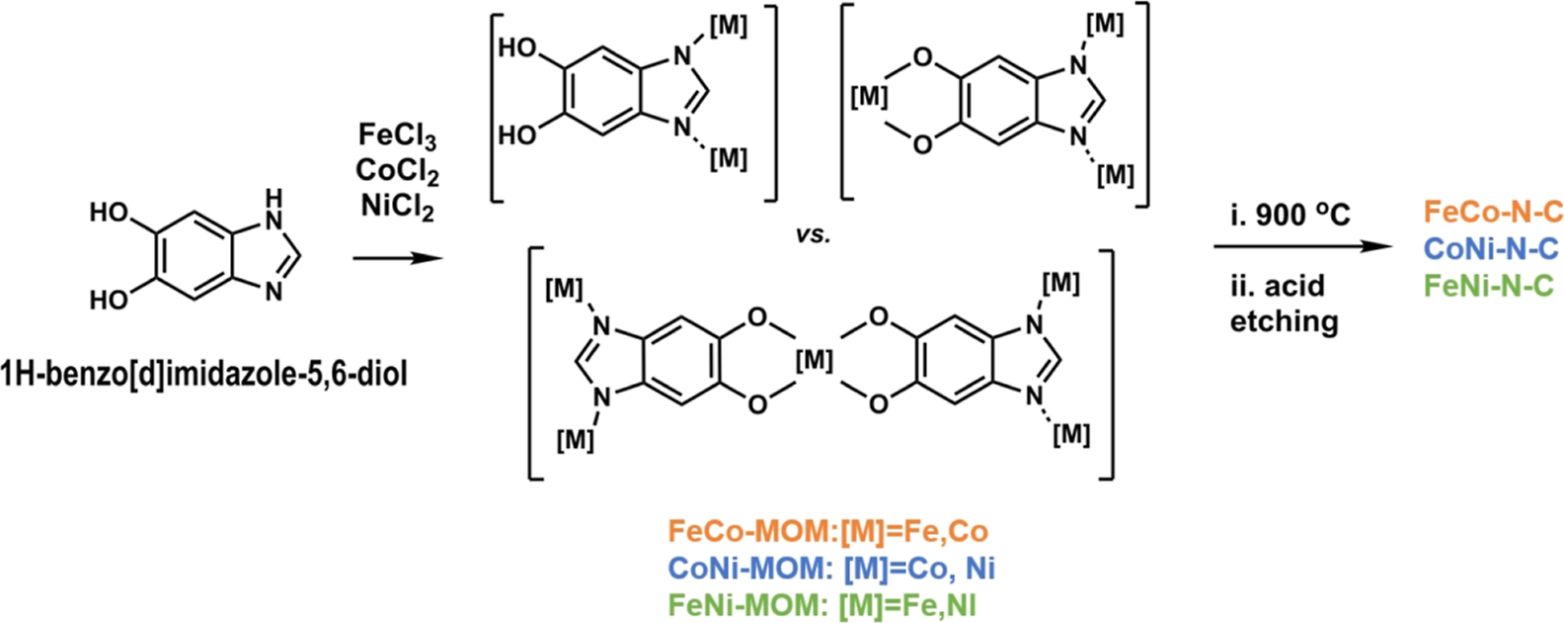
Synthesis of Bimetallic M–N–C Catalyst Materials

Bimetallic M–N–C catalysts were synthesized by carbonizing metal organic materials containing Fe and Co, or Fe and Ni, or Co and Ni. Initially, the organic ligand solutions were mixed with corresponding metal chlorides to form MOMs.^49−51^ Upon carbonization and acid etching of these MOMs, the resulting bimetallic M–N–C catalysts were obtained, as depicted in Scheme 1.

**Figure 1:**
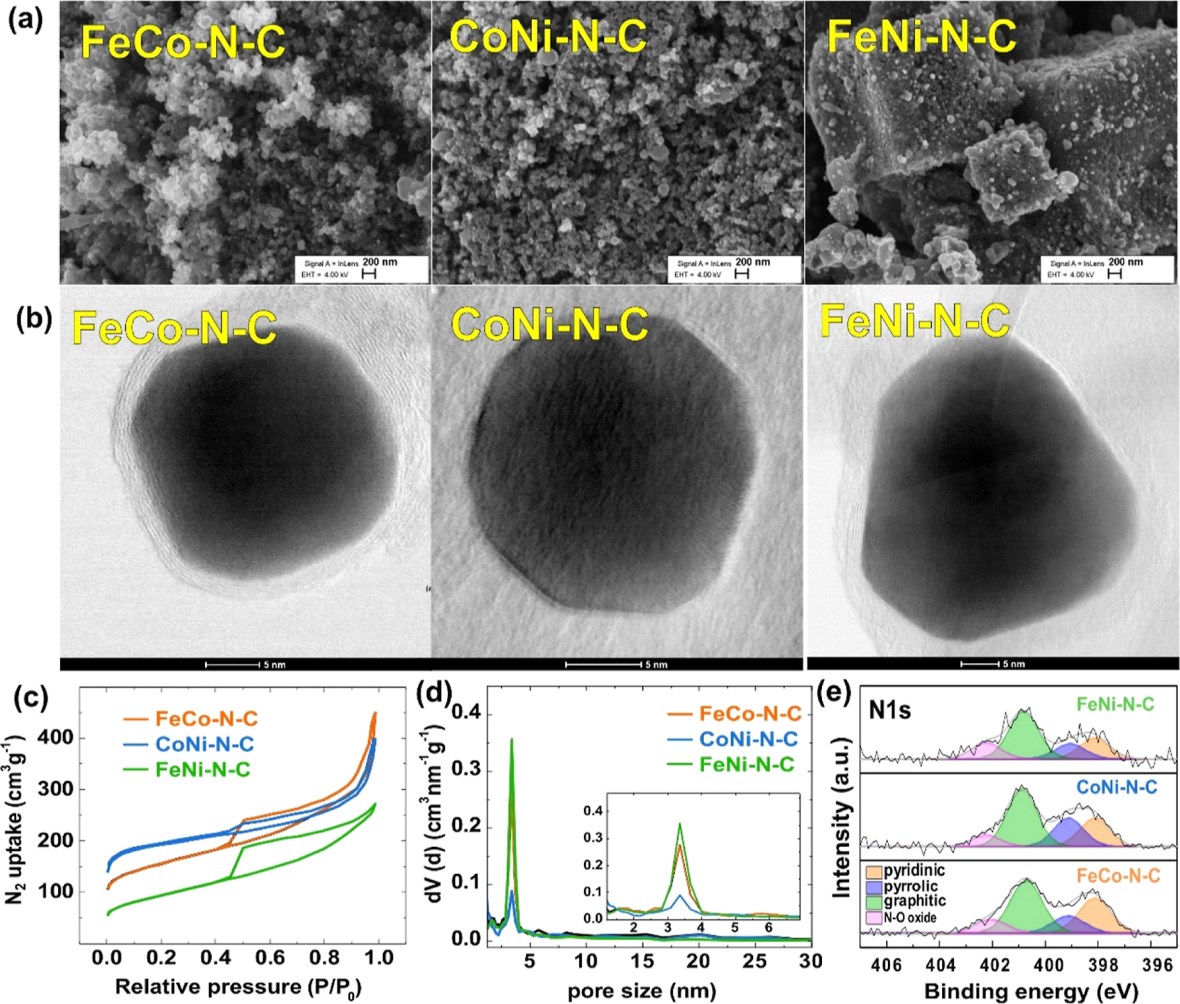
(a) SEM and (b) STEM images of the FeCo–N–C, CoNi–N–C, and FeNi–N–C samples; (c) N_2_ adsorption–desorption isotherms; (d) pore size distribution; and (e) XPS high-resolution spectra in the N 1s region.

The SEM and STEM analyses clearly indicated that, following the processes of carbonization and acid etching, the catalyst materials exhibited a distinct characteristic—nanoparticles of diverse dimensions seamlessly embedded within the carbon support (as illustrated in Figures 1a,b, and S1). The carbon support is predominantly in the form of amorphous carbon, fostering electrolyte diffusion and electron transport by providing abundant defects.^54^ The nitrogen adsorption–desorption isotherms indicate that the materials exhibited a high degree of porosity, with typical pore sizes of 3–4 nm, as illustrated in Figure 1c,d. Particularly, the FeNi–N–C sample displayed a higher mesopore content compared to the other two samples, a characteristic that enhances ion penetration and mass transfer.^55^ The quantitative results of the BET analysis are summarized in Table S2, revealing important insights into the porosity of the catalyst materials under study. CoNi–N–C exhibited the highest specific surface area of 673 m^2^ g^–1^, compared to FeCo–N–C and FeNi–N–C samples (545 and 332 m^2^ g^–1^, respectively). The results of BET analysis were further confirmed by EIS measurements (Figure S3). According to the EIS results, CoNi–N–C has the lowest charge-transfer resistance among FeCo–N–C and FeNi–N–C, suggesting its fastest electron transfer rate.

XPS survey spectra revealed the presence of corresponding metals at the surface of catalysts (Figure S2a), which was further confirmed by HAADF-STEM elemental mapping. The atomic distribution of elements on the surface of the catalysts, as determined by XPS, is presented in Table S3. An analysis of the high-resolution XPS spectra in the N 1s region (Figure 1e) revealed that all three catalyst materials exhibited a high percentage of pyridinic and graphitic nitrogen (398.9 and 400.8 eV, respectively), as well as pyrrolic-N (399.5 eV) and oxidized-N (404.6 eV). The obtained percentage of each nitrogen species is summarized in Table S4.

As shown in Figure S2c, the high-resolution Fe 2p XPS spectrum of the FeNi–N–C sample shows peaks corresponding to the single-atomic Fe–N_*x*_ species formed during the pyrolysis process.^56^ Therefore, it can be concluded that in the FeNi–N–C sample, the Fe single-atomic sites coexist with nanoparticles, which can benefit oxygen electrocatalysis. The results of the XRD analysis provided important information about the crystalline components present in the samples under study. The XRD patterns, as shown in Figure S2b, revealed the presence of specific metal alloys in each of the samples. The FeCo–N–C sample was found to contain α-FeCo nanoparticles, and the CoNi–N–C sample contained CoNi nanoparticles. In the FeNi–N–C sample, both α-Fe and FeNi nanoparticles were detected, indicating that the samples contained a mixture of different phases. The multiphase composition might substantially affect the electrocatalytic properties.

To quantify the overall contents of the different metals within a sample, the MP-AES technique was used. Table S5 lists the results of the MP-AES analysis. Even though all MOMs were prepared using a 1:1 ratio of the metal precursors, the ratio changed for the final. FeCo–N–C contained Fe and Co indeed in a 1:1 ratio, while CoNi–N–C contained Co and Ni in a 1:2 ratio and FeNi–N–C contained Fe and Ni in a ratio of 3:1.

**Figure 2:**
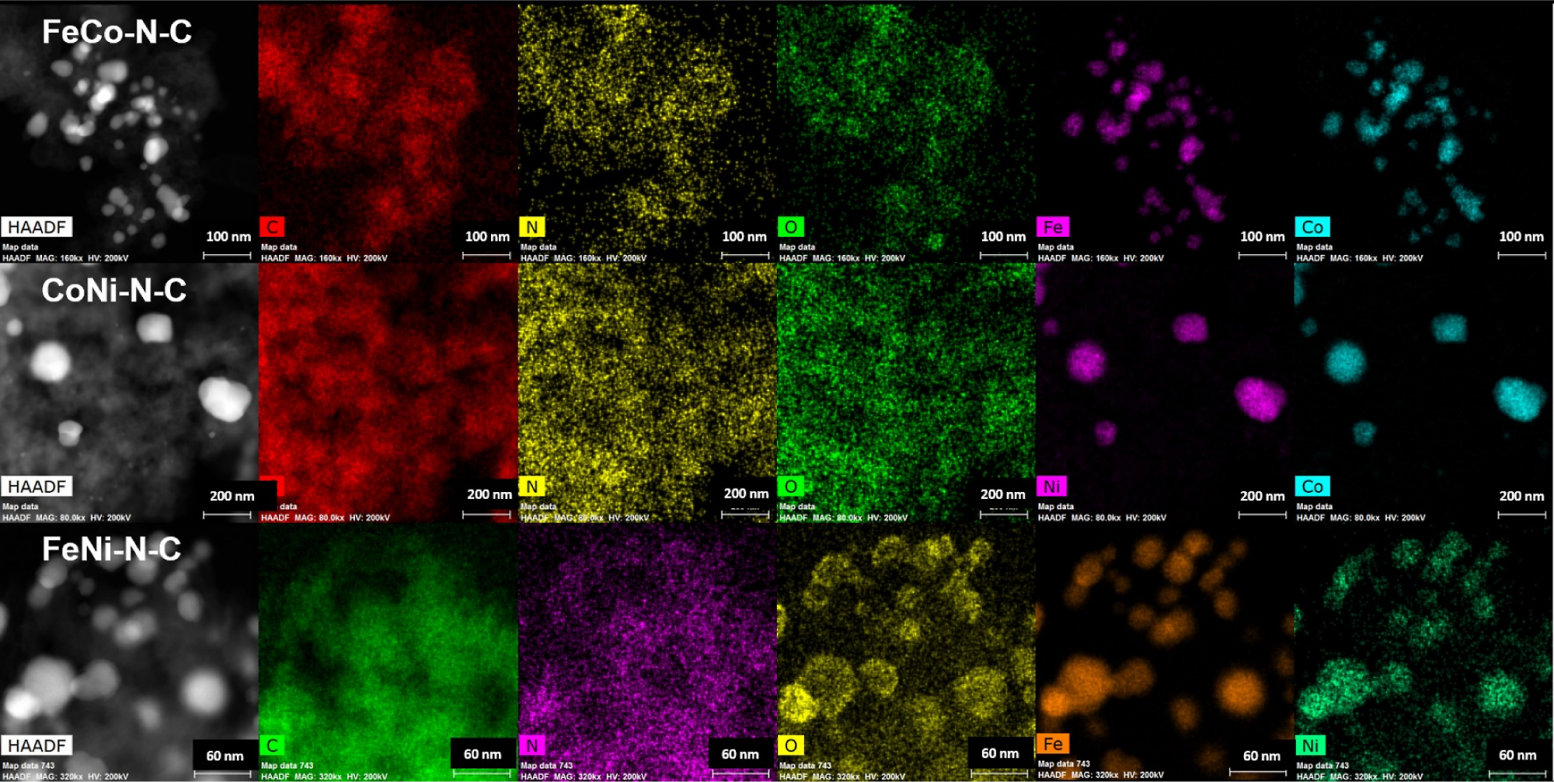
Elemental mapping of bimetallic M–N–C catalyst materials using HAADF-STEM.

The HAADF-STEM micrographs (Figure 2) vividly demonstrate the uniform colocalization of metals across all of the analyzed samples. This is an important observation as it indicates that the metals are likely interacting with each other and forming alloy nanoparticles, as opposed to remaining as separate, distinct entities. The presence of these metal alloy nanoparticles of various compositions is an early indication that our synthetic approach was successful and in agreement with XRD analysis. The well-dispersed distribution of nitrogen and oxygen within the carbon networks also indicates that the synthetic approach employed in this study is effective in forming a new class of bimetallic M–N–C materials. While the concentration of transition metals and oxygen elements may appear more pronounced in certain regions, this can be attributed to the presence of metal oxides, which coexist with the M–N–C structure. The distribution of both nitrogen and oxygen at the surface of the catalyst varied depending on the sample, ranging from 0.93 to 2.43% (Table S3).

### 3.2 Electrocatalytic Performance of Bimetallic M–N–C Materials

**Figure 3:**
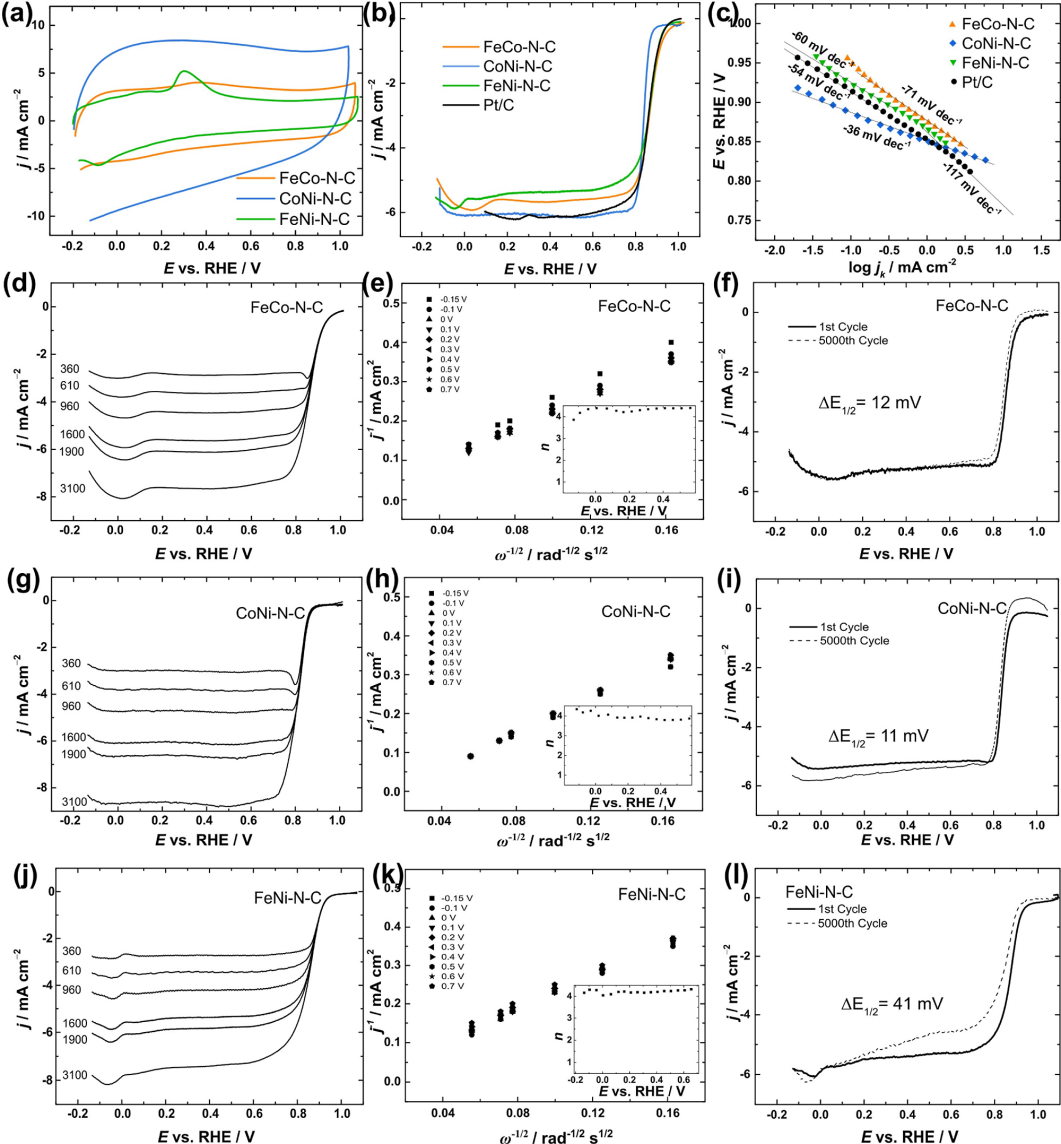
(a) CV curves obtained for bimetallic M–N–C catalyst materials in Ar-saturated 0.1 M KOH at a scan rate of 50 mV s^–1^; (b) comparison of RDE voltammetry curves for oxygen reduction obtained for all catalysts in O_2_-saturated 0.1 M KOH at ω = 1600 rpm and ν = 10 mV s^–1^; (c) comparison of Tafel plots constructed from the RDE data; (d,g,j) ORR polarization curves recorded for M–N–C catalysts at different rotation rates, ν = 10 mV s^–1^; (e,h,k) Koutecky–Levich plots derived from the RDE data (insets: *n* values as a function of potential); and (f,i,l) ORR polarization curves recorded before and after long-term potential cycling.

The ORR performance of synthesized bimetallic M–N–C materials, as well as the commercial Pt/C benchmark, was assessed in 0.1 M KOH solution in a three-electrode system using an RDE setup. Figure 3a shows the CV curves recorded for all three catalysts in an Ar-saturated electrolyte at a scan rate of 50 mV s^–1^. CV profiles reveal characteristic electric double layer formation across all tested M–N–C samples, indicative of their capacitive behavior. Notably, the CoNi–N–C electrode exhibits a pronounced increase of the charging current of the double layer, which can be attributed to a higher accessible surface area, confirmed by the specific surface area (*S*_BET_) of 673 m^2^ g^–1^. The pore structure of the catalysts becomes as much more open as more metal is removed. The FeNi–N–C sample displays the lowest current, characterized by two peaks—an anodic peak at 0.30 V and a cathodic peak at −0.1 V vs RHE. CV results are in accordance with BET analysis results, which revealed the lowest *S*_BET_ of 332 m^2^ g^–1^ and a smallest total pore volume of 0.387 cm^3^ g^–1^ for the FeNi–N–C catalyst. What in combination with EDX mapping and overall much higher Fe content would point to a less effective acid leaching compared to the other samples.

To further assess the electrocatalytic activity of the prepared catalysts in both ORR and the OER, RDE measurements were performed at an electrode rotational speed of 1600 rpm in an O_2_-saturated electrolyte (Figure 3b). Both FeNi–N–C and FeCo–N–C catalysts displayed superior ORR activity compared to CoNi–N–C as demonstrated by their higher onset potentials (*E*_on_ = 1.02 V) and half-wave potentials (*E*_1/2_ = 0.86 V). Notably, the kinetic parameters obtained for FeNi–N–C and FeCo–N–C were found to be on par with those obtained for a commercial Pt/C catalyst (*E*_on_ = 1.01 V; *E*_1/2_ = 0.85 V, Table 1) and to be superior to previously reported bimetallic M–N–C catalyst materials (Table S7). The exceptional performance of FeCo–N–C material results from the combination of FeCo alloy nanoparticles and diverse M–N_*x*_ active sites, which facilitate rapid oxygen adsorption/desorption and significantly enhance reaction efficiency.^57^ The simultaneous presence of both Fe and Ni nanoparticles within the FeNi–N–C catalyst was also confirmed to greatly enhance the ORR process.^58^ The remarkable ORR performance of FeCo–N–C can also be ascribed to the increased proportion of pyridinic nitrogen species, contributing to the efficiency of ORR electrocatalysis.^59^

**Table 1:** Comparison of the ORR and the OER Kinetic Parameters Obtained for Different Catalysts in 0.1 M KOH

catalyst	*E*_1/2_ (V vs RHE)	*E*_on_ (V vs RHE)	*E*_*j*=10_ (V vs RHE)	η_OER_ (V)	Δ*E* (V)
FeCo–N–C	0.86	1.02	1.59	0.36	0.73
CoNi–N–C	0.85	0.96	1.58	0.35	0.73
FeNi–N–C	0.86	1.02	1.58	0.35	0.72
Pt/C + RuO_2_	0.85	1.01	1.69	0.46	0.84

Despite the fact that the FeNi–N–C sample had the lowest specific surface area, as shown in Table S2, the superior ORR performance of FeNi–N–C may indicate a higher density of electrochemically accessible ORR-active sites and improved mass transfer due to a more appropriate mesoporous structure,^60^ as observed in Figure 1d. The presence of mesopores in all three materials facilitates the diffusion of reactants and products and may account for high ORR performance. The Tafel analyses (Figure 3c) demonstrated that the FeNi–N–C material had a Tafel slope value of −60 mV dec^–1^, which was the closest to that of Pt/C, indicating that the rate-determining step for the ORR is the first electron transfer step. The Koutecky–Levich (K–L) plots were constructed from the RDE data shown in Figure 3e,h,k and the calculated number of electrons transferred per O_2_ molecule (*n*) was close to four for all catalysts in this study (see insets in Figure 3e,h,k).

Continuous potential cycling in the range between 0.2 and 1.2 V was used to assess the long-term stability of prepared M–N–C electrocatalysts with respect to the ORR. All three samples exhibited notable stability throughout the evaluation (Figure 3f,i,l). The FeNi–N–C catalyst demonstrated a negative shift of 41 mV in its *E*_1/2_ value after undergoing 5000 cycles (Figure 3l), marking it as the least stable in ORR among the tested catalysts. On the other hand, the remaining catalysts displayed even higher levels of stability, with FeCo–N–C and CoNi–N–C standing out as particularly robust and durable electrocatalysts for the ORR (change in the *E*_1/2_ value of −12 and −11 mV, respectively).

**Figure 4:**
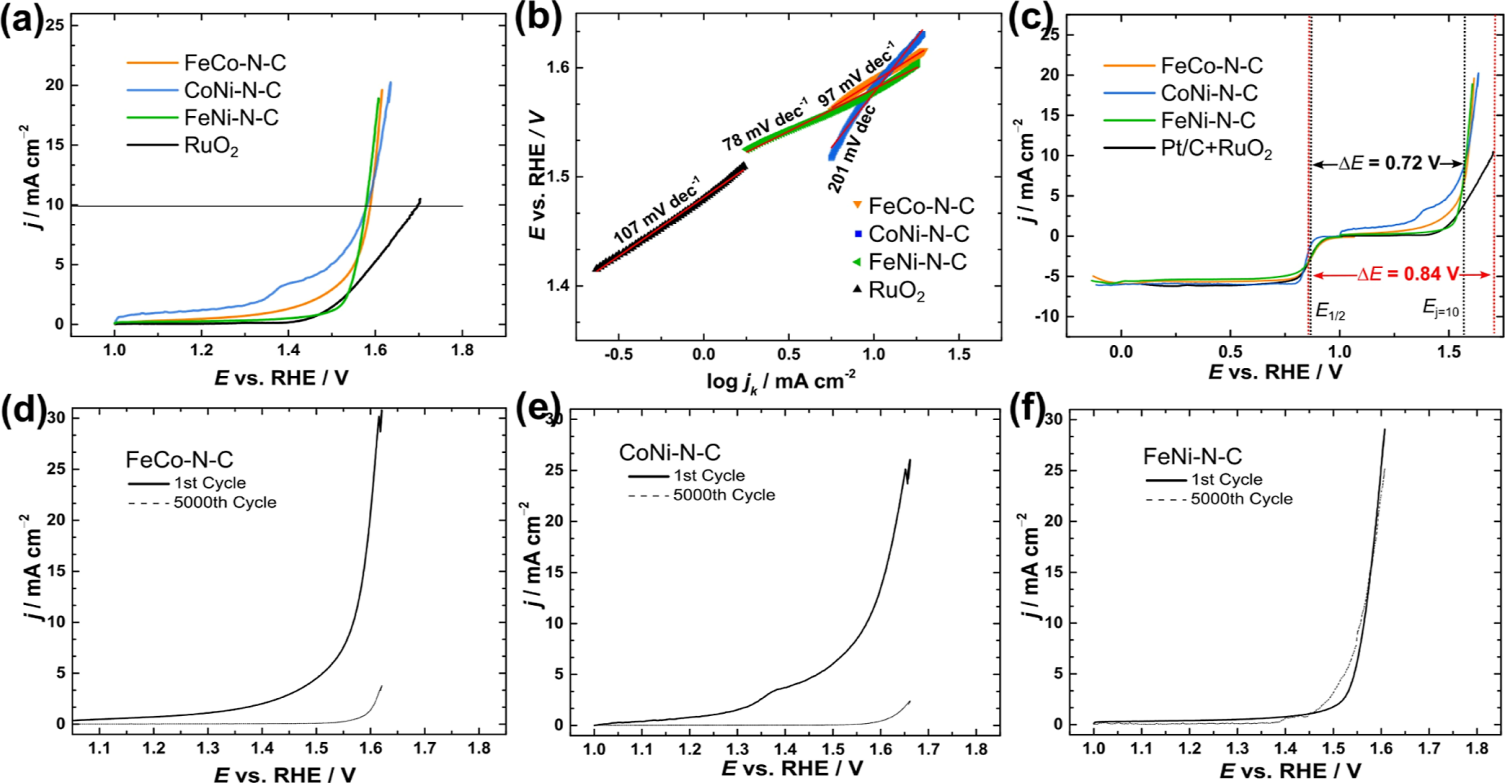
(a) Comparison of OER polarization curves for RuO_2_ and M–N–C catalysts in Ar-saturated 0.1 M KOH at ν = 10 mV s^–1^; (b) comparison of Tafel plots constructed from the OER data; (c) comparison of ORR and OER polarization curves recorded for Pt/C + RuO_2_ and M–N–C catalysts in O_2_-saturated 0.1 M KOH at ν = 10 mV s^–1^; and (d–f) OER polarization curves recorded before and after long-term potential cycling for M–N–C catalysts.

The OER performance of the catalysts was analyzed by using CV and linear sweep voltammetry, and the resulting *iR*-corrected OER polarization curves are presented in Figure 4a. To compare the electrocatalytic activity trends of all studied materials, first, the overpotential required to reach 10 mA cm^–2^ (η_OER_) was calculated and the values are summarized in Table 1. Remarkably, distinctive LSV profiles unveiled an OER overpotential of 360 mV for FeCo–N–C and notably identical OER overpotentials of 350 mV for both CoNi–N–C and FeNi–N–C catalysts. This uniformity in OER overpotential values of the OER indicates comparable bifunctionality of the CoNi–N–C and FeNi–N–C materials. The overpotential values across all of the bimetallic M–N–C catalyst materials consistently outperformed those associated with the commercial ruthenium oxide benchmark (460 mV), signifying the potential superiority of these studied catalysts in oxygen evolution applications.

The enhanced OER performance of bimetallic M–N–C electrocatalysts is caused by the synergy of complementary metals.^61,62^ Moreover, the optimized micro- and mesoporosity of the M–N–C catalysts promote electrolyte penetration and expose more active sites for the OER. The FeNi–N–C catalyst exhibited the lowest overall oxygen bifunctional electroactivity value (Δ*E*) of 0.72 V, as shown in Figure 4c and Table 1. The OER Tafel plots were constructed from the LSV data (Figure 4b) and obtained Tafel slope values confirmed that bimetallic M–N–C catalysts outperformed the commercial RuO_2_ benchmark. It was proposed that lower Tafel slope values might be associated with decreasing overpotentials, thus faster kinetics of the OER.^63^ However, the exact mechanism of the OER at bimetallic catalyst surfaces is still in question. Recently, Zhang et al. proposed that OER active sites on bimetallic NiFe–N–C are created through a multifaceted process, where the exposed Ni_2_Fe_1_ alloy in Ni_2_Fe_1_@PANI-KOH900 transforms into Ni(Fe)OOH particles under OER conditions, known for their excellent OER catalytic activity.^64^ Recently, Meng et al. synthesized a dual-metal NiFe–N–C catalyst with atomically dispersed Ni–N_4_ and Fe–N_4_ sites alongside adjacent Fe nanoclusters which exhibited exceptional bifunctional activity with an ultrasmall Δ*E* of 0.68 V and negligible decay of key parameters after extensive cycling.^65^ In another work by Wang et al., a highly active bifunctional FeNi_AC_-NC catalyst was prepared by the pyrolysis of phenanthroline, activated carbon, ferrocene, and nickelocene.^66^ Obtained bimetallic cluster catalysts exhibited high ORR activity with *E*_1/2_ = 0.936 V due to the synergistic effect of uniformly dispersed Fe/Ni diatomic clusters and larger Fe/Ni nanoclusters on the N-doped carbon substrate.

The OER performance of the catalysts was tested during long-term potential cycling in a range from 1.0 to 1.8 V vs RHE at room temperature in 0.1 M KOH (Figure 4d,e). Under long-term cycling, the FeNi–N–C material exhibited the highest durability (Figure 4f). It can be assumed that the initially metallic surface gets oxidized under these conditions forming oxyhydroxide-terminated surfaces which are active for the OER.^67^ As summarized by McCrory et al., catalysts containing nickel and iron outperformed any other combination of PGM-free catalysts.^68^ The high stability of bimetallic FeNi-based catalysts was also observed in earlier reports^69,70^ and is generally associated with forming Fe and Ni nitrogen-ligated sites at the catalyst surface after carbonization at 900 °C.

### 3.3 Aqueous Zinc–Air Battery Tests

**Figure 5:**
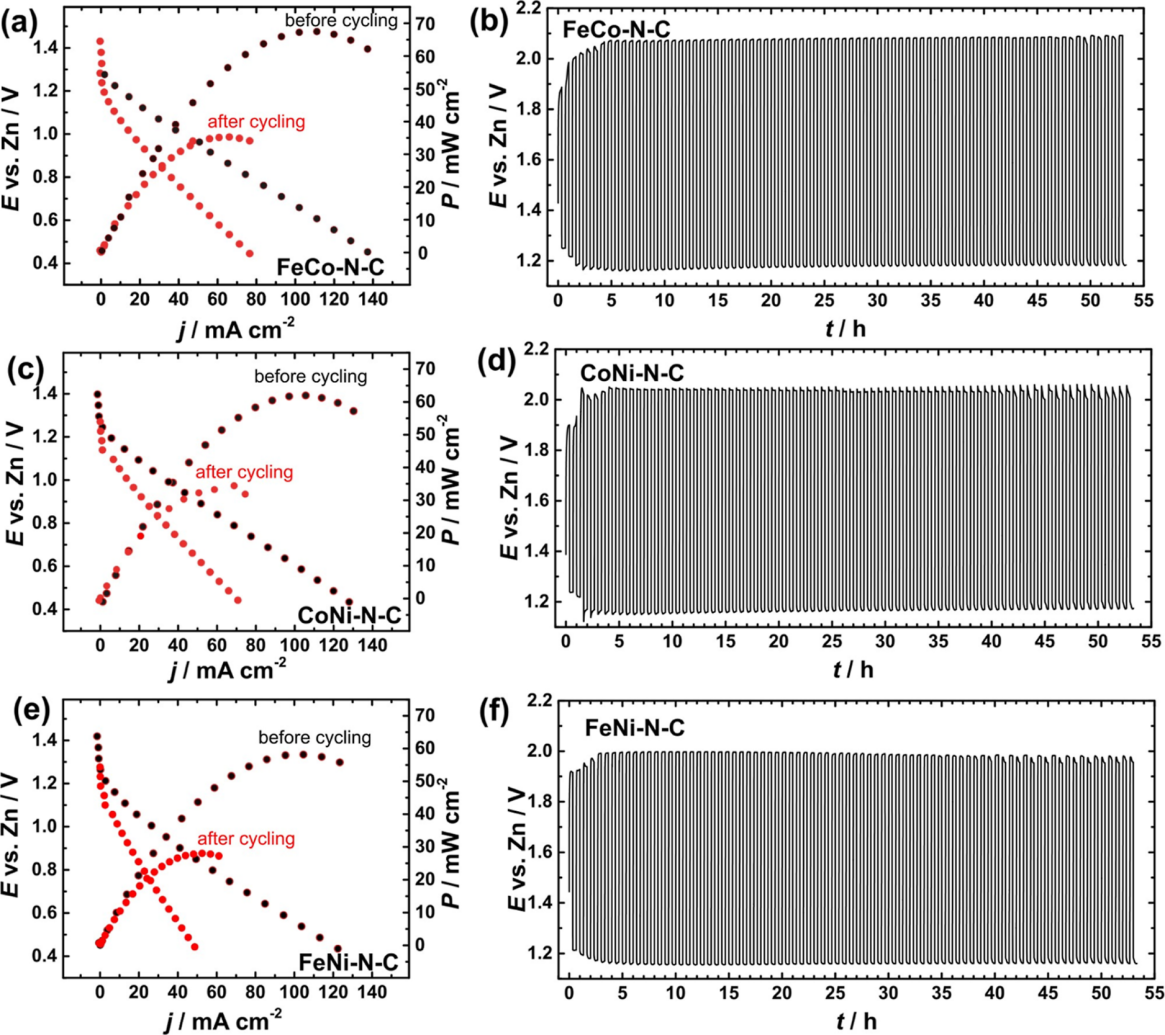
(a,c,e) Polarization and power density curves (scan rate of 1 mV s^–1^) and (b,d,f) charge/discharge cycling results (at 2 mA cm^–2^) obtained for bimetallic M–N–C catalysts.

Aqueous zinc–air battery tests were conducted to evaluate the performance of bimetallic M–N–C catalysts (Figure 5). The ZABs showcased an open-circuit voltage spanning from 1.43 to 1.45 V (Figure S4) and an impressive peak power density (60, 65, and 70 mW cm^–2^ for FeNi–N–C, CoNi–N–C, and FeCo–N–C, respectively) notably surpassing the value obtained for the commercial Pt/C + RuO_2_ catalyst under the same conditions (50 mW cm^–2^). The polarization curves, coupled with the charging and discharging cycles of the ZAB depicted in Figure 5, distinctly indicate the superior performance of all bimetallic M–N–C catalysts when compared to the Pt–Ru/C benchmark (Figure S5). The ZAB performance of the catalysts investigated in this study is lower compared to the exceptional outcomes for FeNi–N–C catalysts reported in very recent works.^65,66^ However, the referenced studies employed multiple precursors for the synthesis of catalysts, such as a two-step pyrolysis process or copyrolysis of various compounds. In contrast, the methodology adopted in our investigation involved the utilization of single-precursor bimetallic MOMs. Nevertheless, bimetallic M–N–C catalyst materials presented in this work show promising overall oxygen bifunctionality, as elucidated by the comparative data provided in Table S7.

For a thorough assessment, polarization curves were acquired both prior to and after the charging and discharging cycles, offering valuable insights into the extent of performance decline in the battery. Across all samples, there was an initial rise in the round-trip potential between oxidation and reduction within the initial 5 h period, followed by a phase of sustained stability. After this stabilization period, the performance exhibited consistency throughout the remaining operational duration. The graphical representations of the round-trip efficiency (*E*_charge_ – *E*_discharge_) and voltaic efficiency (*E*_charge_/*E*_discharge_) are provided in Figure S6. According to obtained ZAB key performance parameters such as open circuit potential, round-trip efficiency, and stability, it can be concluded that bimetallic iron triad M–N–C catalysts developed in this work can be promising candidates for scaling up in practical applications.

## 4 Conclusions

In this work, bimetallic iron triad-based M–N–C catalyst materials were successfully synthesized by incorporating carbon-embedded nanoparticles with diverse compositions. This was achieved by carbonizing singular precursors, a novel bimetallic MOM synthesized from 1*H*-benzo[*d*]imidazole-5,6-diol. All of the prepared catalysts exhibited notably high bifunctional oxygen electroactivity. Following a comprehensive assessment of crucial kinetic parameters, including *E*_1/2_, *E*_on_, *E*_*j*=10_, η_OER_, and Δ*E*, FeNi–N–C demonstrated comparable performance to FeCo–N–C and CoNi–N–C; however, it notably outperformed them in terms of long-term stability, particularly evident after 5000 potential cycles under harsh OER conditions, exhibiting minimal performance degradation. The advantageous role of nickel, potentially linked to the creation of nickel (oxy)hydroxide known for its strong intrinsic activity in the OER, likely contributed to this outcome. Upon comprehensive assessment, accounting for ZAB key parameters—open-circuit potential, round-trip efficiency, and stability—the designed bimetallic iron triad M–N–C catalysts show promise for practical application.

## Supporting Information Available

Additional experimental details; particle size distribution histograms; XPS spectra; XRD patterns; electrochemical impedance spectra; additional ZAB experiments; synthesis product yields; data obtained from BET analysis; data obtained from XPS and MP-AES analysis; and performance comparison (PDF)
