# Supplementary material for: Iron Triad-Based Bimetallic M–N–C Nanomaterials as Highly Active Bifunctional Oxygen Electrocatalysts
Source: ACS Appl Energy Mater. 2024 May 2;7(9):4076–87. doi: 10.1021/acsaem.4c00366 (PMC11095250; doi:10.1021/acsaem.4c00366)
Supplement: Supplementary file 1 — ae4c00366_si_001.pdf [file ae4c00366_si_001.pdf]

Supporting Information

for

Iron Triad Based Bimetallic M-N-C Nanomaterials as Highly Active Bifunctional Oxygen Electrocatalysts

Mahboob Alam, [a,f] Kefeng Ping,[a] Mati Danilson,[b] Valdek Mikli,[b] Maike Käärik,[c] Jaan Leis,[c] Jaan Aruväli,[d] Päärn Paiste,[d] Mihkel Rähn,[e] Väino Sammelselg,[e] Kaido Tammeveski,[c] Steffen Haller,[f] Ulrike I. Kramm,[f] Pavel Starkov[a], Nadezda Kongi[c*]

[a]*Department of Chemistry and Biotechnology, Tallinn University of Technology, 12618 Tallinn, Estonia*

[b]*Department of Materials and Environmental Technology, Tallinn University of Technology, 19086 Tallinn, Estonia*

[c]*Institute of Chemistry, University of Tartu, 50411 Tartu, Estonia*

[d]*Institute of Ecology and Earth Sciences, University of Tartu, 50411 Tartu, Estonia*

[e]*Institute of Physics, University of Tartu, 50411 Tartu, Estonia*

[f]*Department of Chemistry, Catalysts and Electrocatalysts Group, Technical University of Darmstadt, 64287 Darmstadt, Germany*

Corresponding authors: nadezda.kongi@ut.ee

Electrochemical measurements

Electrochemical impedance spectra (EIS) were performed under the frequency of 10^2 - 10^6 Hz in 0.1 M KOH. EIS was used to determine the resistance (*R*) which was in the range from 40 to 50 Ω. All the measurements were *i*R–corrected using built in electrochemical impedance spectroscopy tool using NOVA software.

ORR Koutecky–Levich (K–L) plots at different potentials were plotted to find out the number of electrons transferred per O_2 molecule using RDE data by putting values in the following equation.

$$\frac{1}{j}=\frac{1}{j^{\mathrm{k}}}+\frac{1}{j^{\mathrm{d}}}=-\frac{1}{nFkC_{O^2}^{b}}-\frac{1}{0.62nFD_{O^2}^{2/3}\nu^{-1/6}C_{O^2}^{b}\omega^{1/2}}$$

where j, j_k, j_d denote the measured current density at a specified potential, kinetic current density and diffusion limited current density, respectively. *n* is the number of electrons transferred per O_2 molecule, *F* is the Faraday constant (96,485 C mol^{-1}), *k* is the electrochemical rate constant for O_2 reduction reaction (unit: cm s^{-1}), $C_{O2}{}^{b}$ is the concentration of oxygen in the bulk (1.2×10^{-6} mol cm^{-3}), D_{O2} is the diffusion coefficient of O_2 (1.9×10^{-5} cm^2 s^{-1}) and v is the kinematic viscosity of the solution (0.01 cm^2 s^{-1}), ω is the rotation rate (unit: rad s^{-1}).

Figures

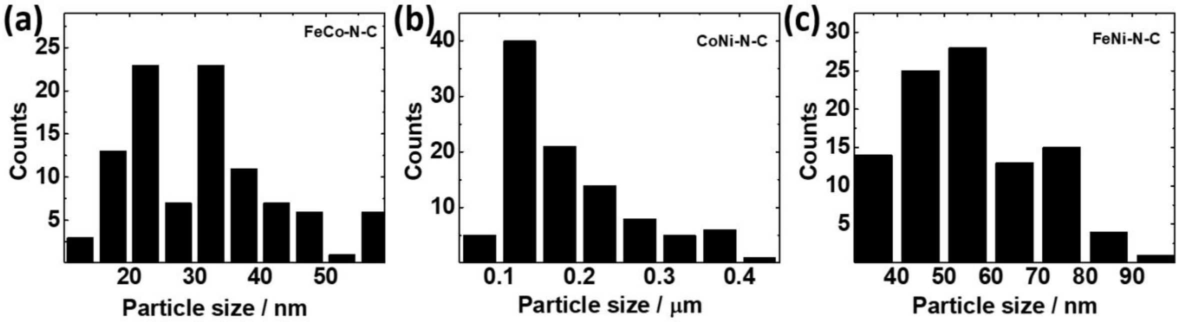

Figure S1. Particle size distribution in prepared bimetallic M-N-C catalyst materials.

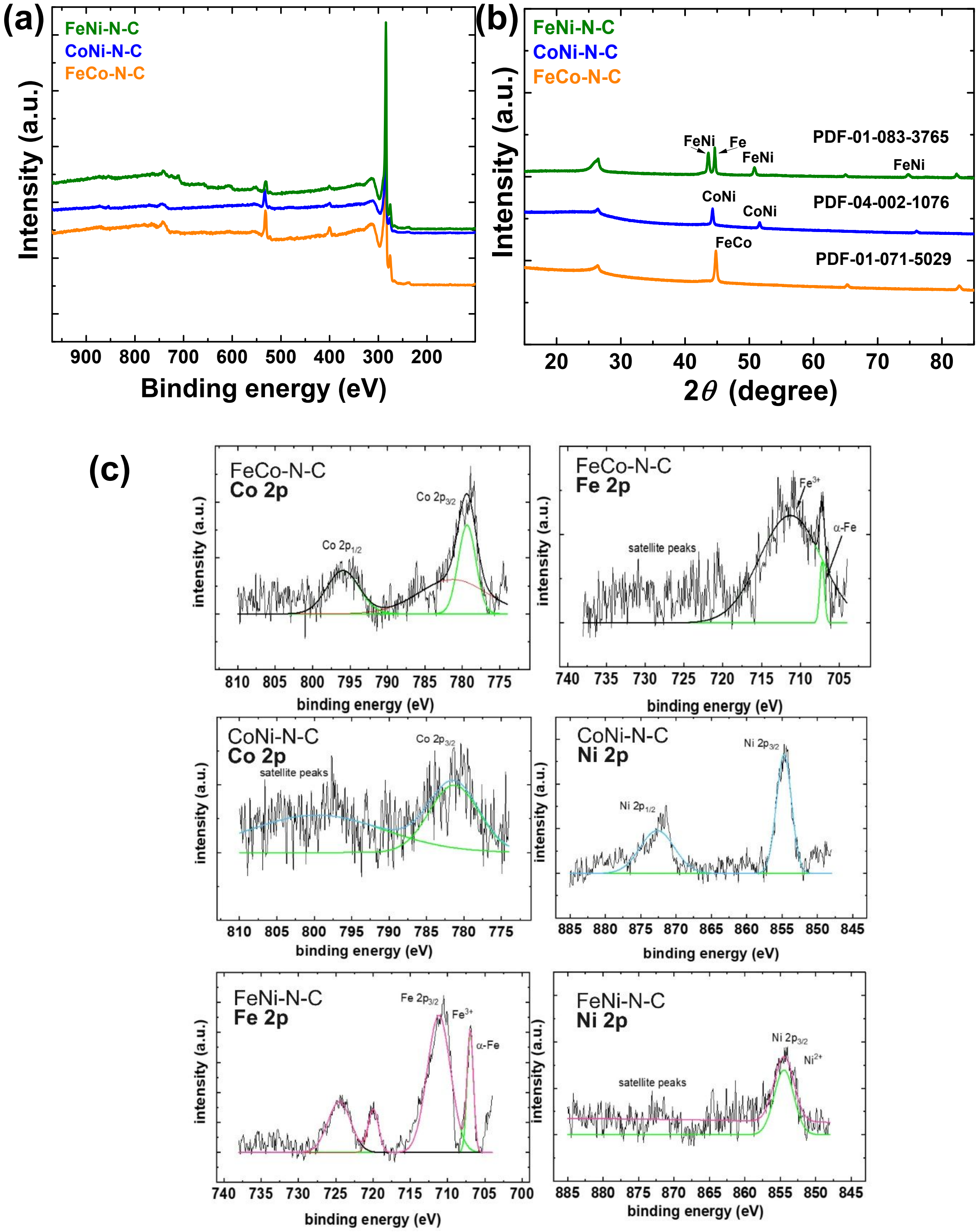

Figure S2. (a) XPS survey spectra of prepared bimetallic M–N–C materials, (b) XRD patterns obtained for bimetallic M–N–C materials (PDFs number are given respective to the color for reference), (c) High resolution Co 2p, Fe 2p an Ni 2p XPS spectra obtained for all bimetallic M-N-C samples.

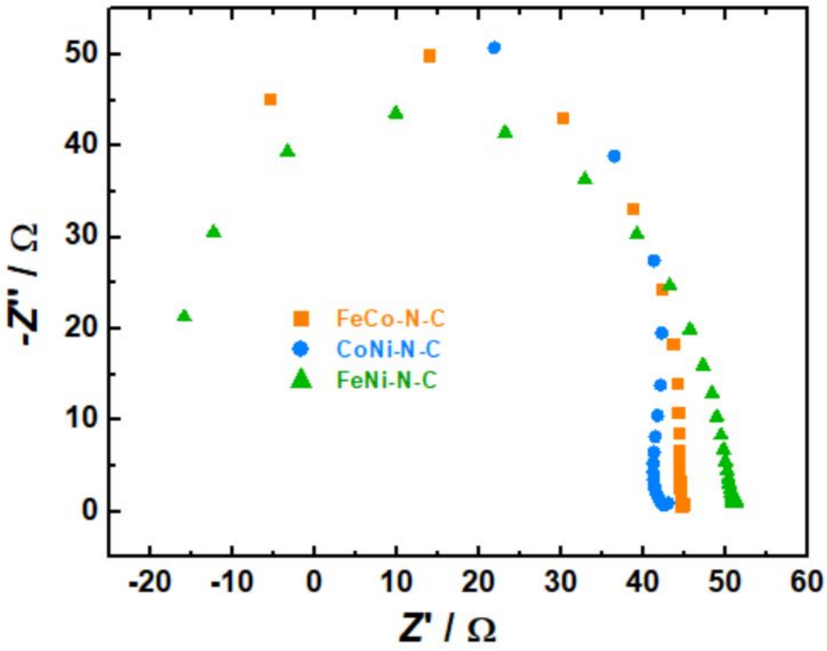

Figure S3. Electrochemical impedance spectra (EIS) of FeCo-N-C, CoNi-N-C and FeNi-N-C measured at frequency range of 102 - 106 Hz in 0.1 M KOH.

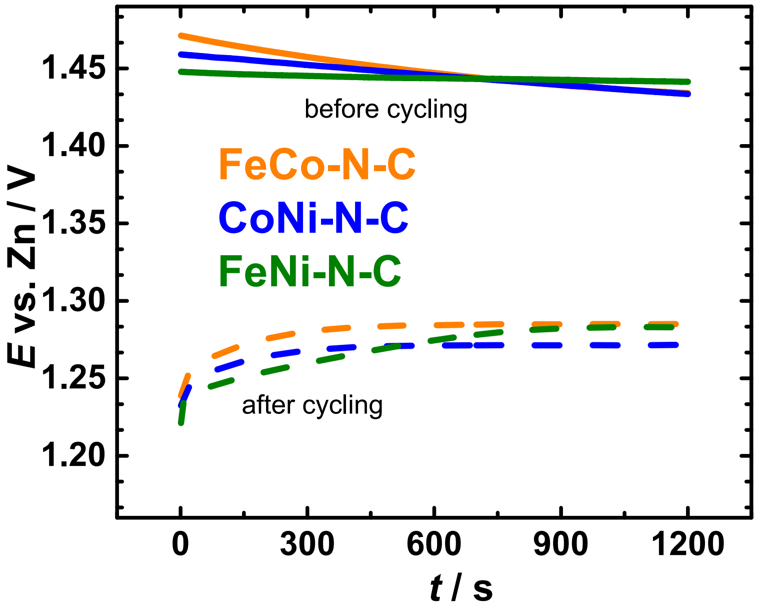

Figure S4. Open circuit potential measured before (solid lines) and after ZAB cycling (dashed lines).

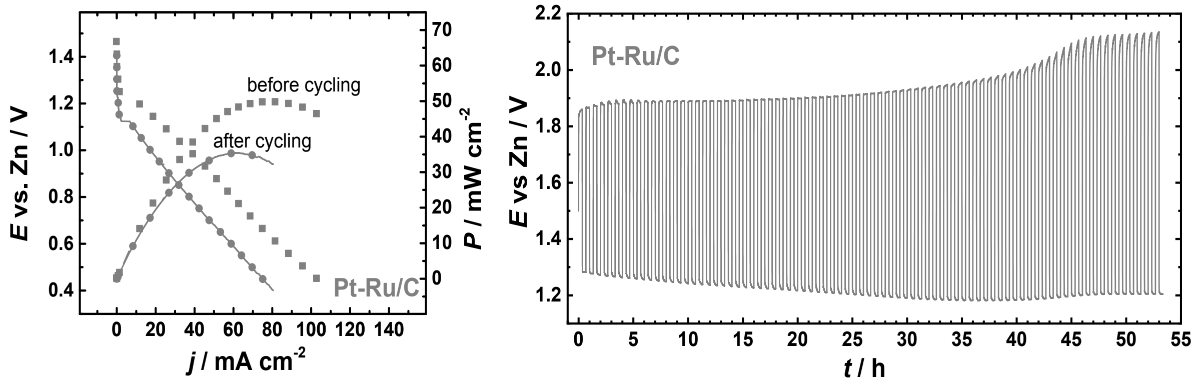

Figure S5. Durability studies of ZAB with commercial Pt–Ru/C catalyst based on polarization and power density curves (1 mV s^{-1}) and charge/discharge cycling results (2 mA cm^{-2}).

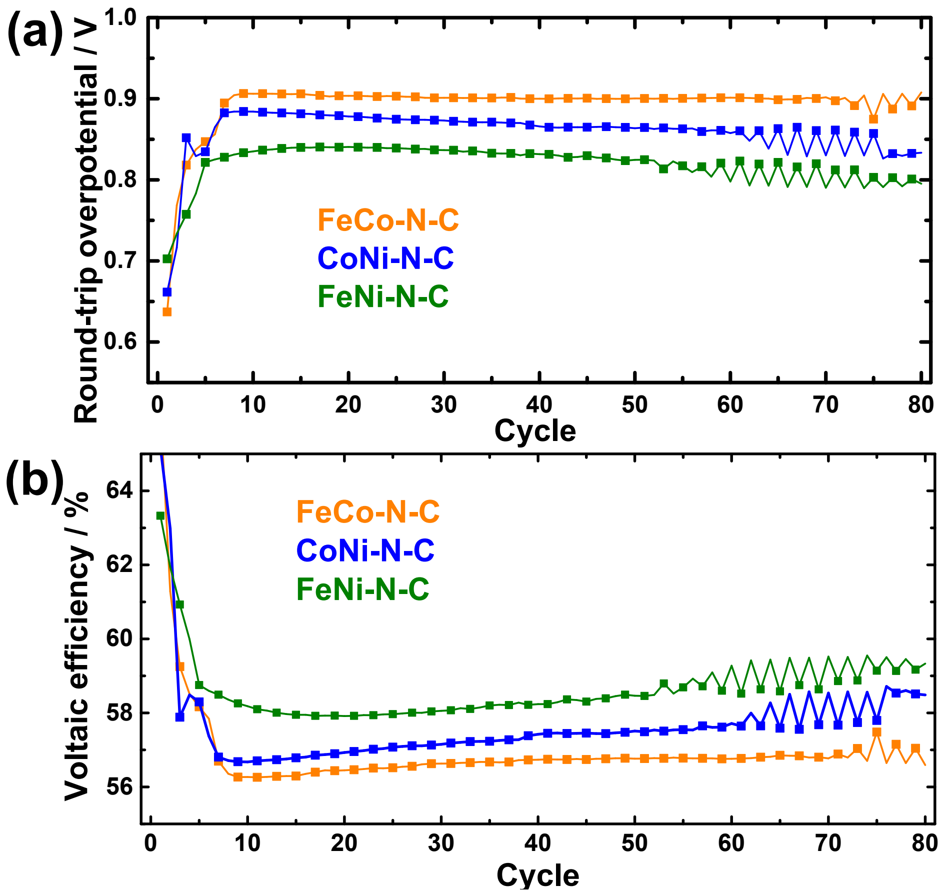

Figure S6. ZAB cycling round-trip efficiency (a), and voltaic efficiency (b) during the set timeframe.

Tables

Table S1. The post-synthetic treatment yields, quantified in milligrams (mg) for each step of the synthesis process. The mass of the used raw material was corrected for the contribution of water/crystal water. For this correction, the yield in metal-(benzimidazole)$_2$ was determined. In case of <100% the pure product was assumed, in case of >100%, it is assumed that the remaining mass can be assigned to water or crystal water.

Catalyst	raw material	1st pyrolysis	yield %	acid leaching	yield %	2nd pyrolysis	yield %
**FeCo-N-C**	1320	460	35 %	400	30 %	280	21 %
**CoNi-N-C**	786	272	35 %	250	32 %	175	22 %
**FeNi-N-C**	1221	447	37 %	388	32 %	271	22 %

Table S2. BET surface area of the prepared catalysts, and total pore and micropore volume.

Catalyst	S_{BET} (m^2 g^{-1})	S_{DFT} (m^2 g^{-1})	V_{tot} (cm^3 g^{-1})	V_{μ} (cm^3 g^{-1})
**FeCo-N-C**	545	559	0.544	0.167
**CoNi-N-C**	673	739	0.526	0.257
**FeNi-N-C**	332	313	0.387	0.076

Table S3. Distribution of elements at the surface of catalyst derived from XPS data.

Catalyst	C at%	N at%	O at%	metal, at%		
				Fe	Co	Ni
**FeCo-N-C**	91.86	2.3	2.06	0.04	0.42	–
**CoNi-N-C**	92.02	2.19	0.93	–	0.09	0.21
**FeNi-N-C**	90.31	0.99	2.43	0.09	–	0.12

Table S4. Distribution of various nitrogen species at the surface of the catalysts (%).

Catalyst	pyridinic N	pyrrolic N	graphitic N	N–O
**FeCo-N-C**	29.6	14.8	44.3	11.3
**CoNi-N-C**	22.8	22.8	45.2	9.1
**FeNi-N-C**	21.1	15.1	47.5	16.1

Table S5. Total metal content in prepared catalyst materials observed by MP–AES.

Catalyst	Fe, wt%	Co, wt%	Ni, wt%
**FeCo-N-C**	4.77 ±0.13	4.54±0.06	
**CoNi-N-C**		2.18±0.01	4.15±0.05
**FeNi-N-C**	14.31±0.05		4.12±0.03

Table S6. Comparison of parameters for the aqueous zinc–air batteries.

**Catalyst**	**Before cycling**		**After cycling**		**Roundtrip efficiency for 54 h V**	**Potential before cycling V**	**Potential after cycling V**
	**CD mA cm^{-2}**	**PD mW cm^{-2}**	**CD mA cm^{-2}**	**PD mW cm^{-2}**			
**FeCo-N-C**	146	68	84	36	0.9	1.43	1.28
**CoNi-N-C**	137	63	80	35	0.87	1.43	1.27
**FeNi-N-C**	132	59	64	28	0.83	1.44	1.28
**Pt–Ru/C**	110	50	81	35	0.93	1.47	1.40

*CD and PD stand for current densities and power densities respectively.

Table S7. Summarized electrochemical parameters from recent articles on FeNi-N-C catalyst materials in alkaline electrolyte.

**Catalyst**	**$E_{1/2}$ (V vs. RHE)**	**E_{on} (V vs. RHE)**	**$E_{j=10}$ (V vs. RHE)**	**η_{OER} (V)**	**ΔE (V)**
FeNi-N-C **(this work)**	0.86	1.01	1.58	0.35	0.72
1.5FeNi@NCNT[1]	0.86	0.95	1.46	0.23	0.6
Fe-enriched-FeNi3/NC[2]	0.79	0.9	1.59	0.36	0.8
FeNi@NC-900[3]	0.84	1.04	1.56	0.33	0.72
FeNi@NC[4]	0.878	-	1.59	0.36	0.712
P-FeNi/NC-900[5]	0.85	-	1.62	0.39	0.77
FeNi@C/NG[6]	0.84	1.0	1.66	0.43	0.82
FeNi/NC[7]	0.81	0.98	1.58	0.35	0.77
Fe/Ni-NC||FeNi@G[8]	0.885	-	1.503	0.273	0.618
FeNi/*N*-GPCM[9]	0.883	-	1.54	0.310	0.657
OPOP-FeNi[10]	0.845	0.965	1.600	0.370	0.755
NiFe–N–C[11]	0.87	-	-	-	0.68
$FeNi_{AC}$-NC[12]	0.936	-	-	-	0.594

References

(1) Wu, M.; Guo, B.; Nie, A.; Liu, R. Tailored Architectures of FeNi Alloy Embedded in N-Doped Carbon as Bifunctional Oxygen Electrocatalyst for Rechargeable Zinc-Air Battery. *J. Colloid Interface Sci.* **2020**, *561*, 585–592. https://doi.org/10.1016/j.jcis.2019.11.033.

(2) Chen, K.; Kim, S.; Rajendiran, R.; Prabakar, K.; Li, G.; Shi, Z.; Jeong, C.; Kang, J.; Li, O. L. Enhancing ORR/OER Active Sites through Lattice Distortion of Fe-Enriched FeNi3 Intermetallic Nanoparticles Doped N-Doped Carbon for High-Performance Rechargeable Zn-Air Battery. *Journal of Colloid and Interface Science* **2021**, *582*, 977–990. https://doi.org/10.1016/j.jcis.2020.08.101.

(3) Deng, S.-Q.; Zhuang, Z.; Zhou, C.-A.; Zheng, H.; Zheng, S.-R.; Yan, W.; Zhang, J. Metal-Organic Framework Derived FeNi Alloy Nanoparticles Embedded in N-Doped Porous Carbon as High-Performance Bifunctional Air-Cathode Catalysts for Rechargeable Zinc-Air Battery. *Journal of Colloid and Interface Science* **2023**, *641*, 265–276. https://doi.org/10.1016/j.jcis.2023.03.073.

(4) Chen, J.; Li, L.; Cheng, Y.; Huang, Y.; Chen, C. Covalent Organic Polymer Derived N–Doped Carbon Confined FeNi Alloys as Bifunctional Oxygen Electrocatalyst for Rechargeable Zinc-Air Battery. *International Journal of Hydrogen Energy* **2022**, *47* (36), 16025–16035. https://doi.org/10.1016/j.ijhydene.2022.03.100.

(5) Yu, T.; Su, S.; Che, Y.; Meng, C.; Zhou, H.; Yan, S.; Patrick Ranson, K.; Bian, T.; Yuan, A. FeNi Alloys Supported on Nitrogen-Enriched Carbon Nanospheres as Efficient Bifunctional Oxygen Electrocatalyst for Rechargeable Zinc-Air Battery. *Journal of Electroanalytical Chemistry* **2023**, *938*, 117433. https://doi.org/10.1016/j.jelechem.2023.117433.

(6) Li, G.-L.; Yang, B.-B.; Xu, X.-C.; Cao, S.; Shi, Y.; Yan, Y.; Song, X.; Hao, C. FeNi Alloy Nanoparticles Encapsulated in Carbon Shells Supported on N-Doped Graphene-Like Carbon as Efficient and Stable Bifunctional Oxygen Electrocatalysts. *Chemistry – A European Journal* **2020**, *26* (13), 2890–2896. https://doi.org/10.1002/chem.201904685.

(7) Li, G.-L.; Xu, X.-C.; Yang, B.-B.; Cao, S.; Wang, X.; Fu, X.; Shi, Y.; Yan, Y.; Song, X.; Hao, C. Micelle-Template Synthesis of a 3D Porous FeNi Alloy and Nitrogen-Codoped Carbon Material as a Bifunctional Oxygen Electrocatalyst. *Electrochimica Acta* **2020**, *331*, 135375. https://doi.org/10.1016/j.electacta.2019.135375.

(8) Xu, Z.; Chen, G.; Yang, F.; Jang, J.; Liu, G.; Xiao, F.; Sun, Y.; Qiu, X.; Chen, W.; Su, D.; Gu, M.; Shao, M. Graphene-Supported Fe/Ni Single Atoms and FeNi Alloy Nanoparticles as Bifunctional Oxygen Electrocatalysts for Rechargeable Zinc-Air Batteries. *Electrochimica Acta* **2023**, *458*, 142549. https://doi.org/10.1016/j.electacta.2023.142549.

(9) Zhang, M.; Hu, X.; Xin, Y.; Wang, L.; Zhou, Z.; Yang, L.; Jiang, J.; Zhang, D. FeNi Coordination Polymer Based Highly Efficient and Durable Bifunction Oxygen Electrocatalyst for Rechargeable Zinc-Air Battery. *Separation and Purification Technology* **2023**, *308*, 122974. https://doi.org/10.1016/j.seppur.2022.122974.

(10) Xiong, Q.; Zheng, J.; Liu, B.; Liu, Y.; Li, H.; Yang, M. In-Situ Self-Templating Construction of FeNi/N Co-Doped 3D Porous Carbon from Bimetallic Ions-Coordinated Porous Organic Polymer for Rechargeable Zinc-Air Batteries. *Applied Catalysis B: Environmental* **2023**, *321*, 122067. https://doi.org/10.1016/j.apcatb.2022.122067.

(11) Meng, H.; Wu, B.; Zhang, D.; Zhu, X.; Luo, S.; You, Y.; Chen, K.; Long, J.; Zhu, J.; Liu, L.; Xi, S.; Petit, T.; Wang, D.; Zhang, X.-M.; Xu, Z. J.; Mai, L. Optimizing Electronic Synergy of Atomically Dispersed Dual-Metal Ni–N4 and Fe–N4 Sites with Adjacent Fe Nanoclusters for High-Efficiency Oxygen Electrocatalysis. *Energy Environ. Sci.* **2024**, *17* (2), 704–716. https://doi.org/10.1039/D3EE03383J.

(12) Wang, Y.; Katyal, N.; Tang, Y.; Li, H.; Shin, K.; Liu, W.; He, R.; Xu, M.; Henkelman, G.; Bao, S.-J. One-Step Pyrolysis Construction of Bimetallic Atom-Cluster Sites for Boosting Bifunctional Catalytic Activity in Zn-Air Batteries. *Small* **2024**, *20* (11), 2306504. https://doi.org/10.1002/smll.202306504.
